# Laser debonding of ultrathin occlusal veneers fabricated from different CAD/CAM ceramic materials

**DOI:** 10.1186/s12903-024-04314-6

**Published:** 2024-05-15

**Authors:** Nourhan Ali El-Sheikh, Marwa Mohamad Wahsh, Ghada Abdelfattah Hussein

**Affiliations:** Department of Fixed Prosthodontics, Faculty of Dentistry, Ain Shams University, African Unity St, Cairo, Egypt

**Keywords:** Laser, Debonding, Ceramics, Ultrathin occlusal veneers, Er;cr:YSGG

## Abstract

**Background:**

Erbium lasers safely offer the possibility of reuse for debonded restorations. Since these lasers have a high affinity for water molecules, they are absorbed by resin cement causing explosive ablation of the cement and thus, the restoration debonds. The efficiency of this process depends on many factors, including the ceramic type, its chemical composition and thickness. Therefore, this study was designed to test the time taken to debond ultrathin occlusal veneers made of three types of milled ceramic materials and evaluate the integrity of these restorations after debonding.

**Methods:**

Three ceramic types were evaluated in this study: lithium disilicate (IPS Emax CAD), highly condensed lithium disilicate (GC initial®LiSi), and translucent zirconia (Katana zirconia STML). Each group consisted of 8 occlusal veneers of 0.5 mm thickness. The samples were cemented to the occlusal surfaces of the upper molar teeth. An Er; Cr: YSGG laser was applied to the occlusal veneers using the scanning method, and time until debonding was calculated. The debonded samples were then inspected under a stereomicroscope for possible damage. Numerical data are presented as the mean with 95% confidence interval (CI), standard deviation (SD), minimum (min.) and maximum (max.) values. Normality and variance homogeneity assumptions were confirmed using Shapiro-Wilk’s and Levene’s tests, respectively. Data were normally distributed and were analyzed using one-way ANOVA followed by Tukey’s post hoc test. The significance level was set at *p* < 0.05 for all tests. Statistical analysis was performed with R statistical analysis software version 4.3.2 for Windows (R Core Team (2023). R: A language and environment for statistical computing. R Foundation for Statistical Computing, Vienna, Austria. URL https://www.R-project.org/).

**Results:**

There was no significant difference in debonding time between the different materials (*p* = 0.995). The longest debonding time was found for Katana STML (87.52 ± 20.45) (seconds), followed by Emax (86.94 ± 20.63) (seconds), while the lowest value was found for LiSi initial (86.14 ± 25.16) (seconds). In terms of damage to the debonded veneers, The Emax and zirconia samples showed no damage. However, 40% of the LiSi samples fractured during debonding, and 20% exhibited cracks. Only 40% of the LiSi samples were sound after debonding.

**Conclusion:**

Er; Cr: YSGG laser can be used efficiently to remove ceramic occlusal veneers. However, its effect on LiSi restorations needs further research.

## Background

Tooth wear may occur due to many factors such as dietary habits, medical conditions, and oral habits. These factors can cause attrition, abrasion, and erosion of the tooth structure [1]. This tooth damage is of great concern to patients regarding their musculoskeletal harmony, occlusal harmony, and esthetics [2, 3]. 

To compensate for and reverse the loss of tooth structure, prosthetic treatment may be indicated. Partial coverage restorations have been considered a conservative treatment option to restore these teeth. Currently, owing to advances in Computer Aided Design/ Computer Aided Manufacturing (CAD/CAM) technology and adhesion protocols, ultrathin occlusal veneers represent a conservative treatment option compared to the extensive treatments used to treat erosive or abrasive tooth loss [4–7]. The materials used for these ultrathin occlusal veneers are ceramics with high strength and fracture toughness to achieve optimum mechanical performance at such thin thicknesses. Lithium disilicate and zirconia fulfill these requirements [8]. The other strategy is to use hybrid ceramics that combine the strength properties of ceramics and the resilience of resin [9, 10]. 

The use of these new partial coverage adhesive restorations mandates the use of meticulous oral hygiene measures. These measures include tooth brushing, flossing, and the use of mouth wash. Chlorhexidine has an anti-bacterial effect; therefore, it is one of the most common mouthwashes used to treat gingivitis and reduce bleeding and plaque accumulation. However, it has side effects such as tooth discoloration. Thus, new anti-discoloration systems have been tested to help reduce discoloration [11]. 

The problem, however, with ultrathin occlusal veneers is their retrieval in cases of fracture, recurrent caries, misplacement, the need for endodontic treatment, or possible discoloration for many reasons such as colored beverages or chlorhexidine mouthwashes [11, 12]. The bonding of occlusal veneers to enamel using resin cement and surface treatments to enhance their bonding make removal quite difficult. The traditional methods for their removal involved grinding of the restoration using diamond burs, which was time-consuming and exhausting for both the patient and the operator; additionally, there was a possibility of iatrogenic damage to the tooth structure [13]. 

Various methods were tested for the removal of all ceramic restorations. Ultrasonic tips reportedly remove all ceramic brackets by directing the bevel of a special ultrasonic tip towards the edge of the bracket. To minimize enamel damage, the residual cement is first removed with the tip then the tip is moved mesiodistally to create a purchase point between the bracket and the tooth. Then, the bond can be broken by applying minimal force with rocking motion [14, 15]. The ultrasonic method didn’t result in ceramic failures. However, the process takes longer time than other techniques [14]. Also, using ultrasonic tips can result in potential periodontal and bone damage due to heat generation. Therefore, ultrasonic tips must be used with proper air and water cooling to avoid raising the temperature of the tooth beyond the critical limit [16]. 

With the introduction of lasers to dentistry and the advances in this field, studies have been conducted on the use of lasers to debond orthodontic brackets, which paved the way for using the same method to debond porcelain veneers and even crowns [17–20]. 

The process of laser debonding occurs through its absorption in resin cement causing resin degradation via three methods: thermal softening, thermal ablation, and photoablation. Thermal ablation is the fastest and most favorable of the three methods since it results in less heat generation [17, 21]. Therefore, compared with other methods, lasers can be employed to debond restorations without much heat generation and in less time.

Erbium lasers (Er: YAG and Er; Cr: YSGG) have a high affinity for water. Their wavelengths (2780 nm and 2940 nm) correspond to the peak of water molecule absorption of laser light. The higher the content of water in a substance is, the greater the absorption will be. Since resin cement has water molecules and residual monomers, the energy of erbium lasers can be readily absorbed and cause explosive ablation at relatively low energy levels [22, 23]. Erbium lasers are also widely available in dental offices due to their wide use with both soft and hard tissues.

The efficiency of laser-assisted ceramic removal depends on many factors such as the ceramic type and chemical composition, shade and opacity, restoration thickness, cement type and shade, and the parameters of the used laser [20, 24, 25]. 

Studies that measured the debonding time of different restorations using laser concluded that debonding time depends on the type and volume of the cement used, the surface area of the restoration, the material of the ceramic restoration, and its thickness [26]. 

Many studies have reported the successful removal of different restorations without damage. Other studies reported the rebonding of the laser-debonded restorations [21]. Despite the success of lasers, there were reports in the literature about changes in the integrity of the removed restoration [27–29]. 

By reviewing the literature regarding laser debonding of ceramics, it seems that more research is needed on the effect of laser radiation on the debonded ceramics and, accordingly, the possibility of reusing them. The need for further research becomes even more pressing with the evolution of new ceramic materials. Therefore, this study aimed to test the effect of Er; Cr: YSGG laser on different ceramic materials used in posterior restorations to determine the difference between them in terms of debonding time and evaluate the effect of laser on these ceramics after debonding.

The null hypothesis stated that there would be no difference in the time of laser debonding of different CAD /CAM materials, nor would there be any change in the integrity of the debonded ultrathin occlusal veneers.

## Methods

A power analysis was designed to have adequate power to apply a statistical test of the null hypothesis that no difference would be found between tested groups. By adopting an alpha level of (0.05) a beta of (0.2), i.e., power = 80%, and an effect size (f) of (0.682) calculated based on the results of a previous study^2^, the predicted sample size (n) was found to be a total of (24) samples (i.e., 8 samples per group). Sample size calculation was performed using G*Power version 3.1.9.7^3^.

Twenty-four freshly extracted sound upper first molars were collected from the clinic of the oral and maxillofacial surgery department at Ain Shams University [ethics committee approval: FDASU-Rec ED012259].

The molars were examined to ensure that they were caries free and had no restorations, and all calculus deposits and soft tissues were removed. Only molars with a 10 mm mesiodistal diameter of the crown were accepted. The collected teeth were stored in saline.

The collected molars were fixed into ready-made plastic molds filled with autopolymerizing acrylic resin (Vertex Dental, 3D systems, The Netherlands.) at approximately 2 mm below the cementoenamel junction. Teeth were positioned in the center of the mold using a paralleling device until curing was completed.

After fixation of the extracted molars in the acrylic blocks, occlusal surfaces are prepared with a blue-coded wheel stone (WR-13 Diaburs Mani, INC Japan (ISO 068/042).

**Fig. 1:**
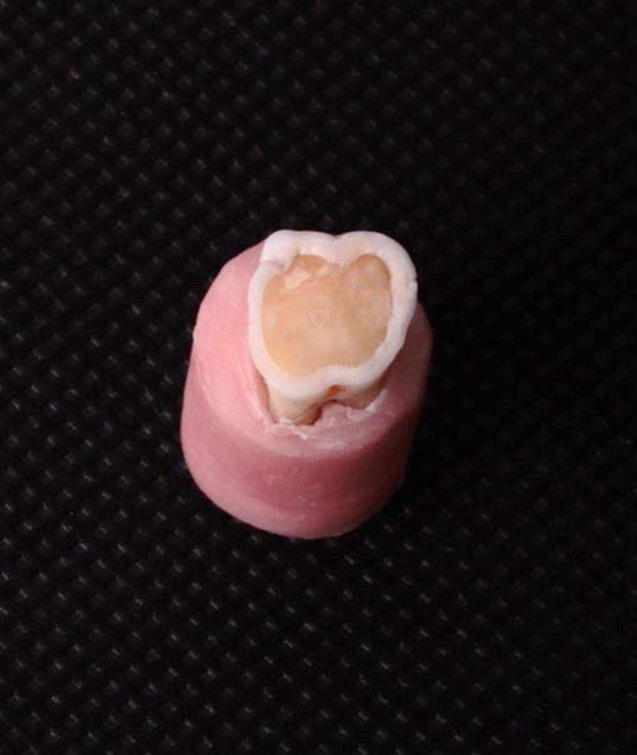
Tooth after preparation. Central dentin and circular enamel was exposed peripherally to simulate a worn occlusal table

The preparation design was a flat occlusal preparation exposing central dentin and circular enamel peripherally at a 4 mm distance from CEJ to simulate a worn occlusal table (Fig. 1).

Preparations were performed under copious irrigation and standardized using a customized surveyor. Each prepared tooth was inspected for disqualifying characteristics such as pulpal exposure, caries, or cracks/fractures.

The prepared teeth were randomly divided into 3 groups according to the material of the ultrathin occlusal veneer: Group A: Lithium disilicate (IPS Emax CAD), Group B: Highly condensed lithium disilicate (GC initial®LiSi), and Group C: Translucent zirconia (STML katana zirconia). Each group consisted of 8 samples (*n* = 8).

Scans of each prepared tooth were obtained using a 3D desktop scanner (Edge, DOF Inc, Korea). The occlusal veneers were designed on CAD software (Exocad GmbH, Germany.) using the Tru Smile technology module. The generic library was used to standardize the design of restorations. The thickness of the restorations was chosen to be 0.5 mm. Finally, the STL (Standard Tessellation Language) files of the final designs were milled by a 5-axis milling machine (350i PRO, imes-icore GmbH, Germany.). Samples were then finished, and the seating on the corresponding teeth was verified. The ceramic samples’ thickness was verified using a digital caliper.

The Prepared Enamel Surfaces were etched with 37% phosphoric acid for 30 s, rinsed with air-water jet for 60 s and dried to remove excess water without desiccation. The glass-ceramic specimens were etched with HF acid (Ceram-etch, Itena, France.) for 20 s and then rinsed thoroughly with water for 60 s to completely remove the etchant and dried well. A Silane coupling agent (Silan It, Itena, France) was then applied to the ceramic surface for 60 s according to the manufacturer instructions. The fitting surface of the zirconia specimens was sandblasted with 50 micron-sized alumina (Al2O3) particles with a pressure of 3.5 bar press for 15 s at a distance of 10 mm. Zirconia MDP primer (Z prime plus, Bisco INC., USA) was applied to the fitting surface and air-dried for 5 s. Cementation of the ceramic samples was performed on the occlusal surface of prepared teeth using a dual-cured resin cement (Breeze™, Pentron clinical technologies, Wallingford, Conn) and a loading device to ensure a uniform cement thickness and to maintain all samples under the same load during cementation (250 gm). Light cure (SmartLite Pro, Dentsply Sirona, USA) with a mean wavelength of 450 nm and an average curing light power 1250 mW/cm^2^ was used to cure the resin cement. Short initial light curing or “tack curing” for 5 s was used to create a semigel state in the luting cement for easier excess material removal, and excess cement was carefully removed at the margins. Curing continued for 20 s at Rapid Mode.

**Fig. 2:**
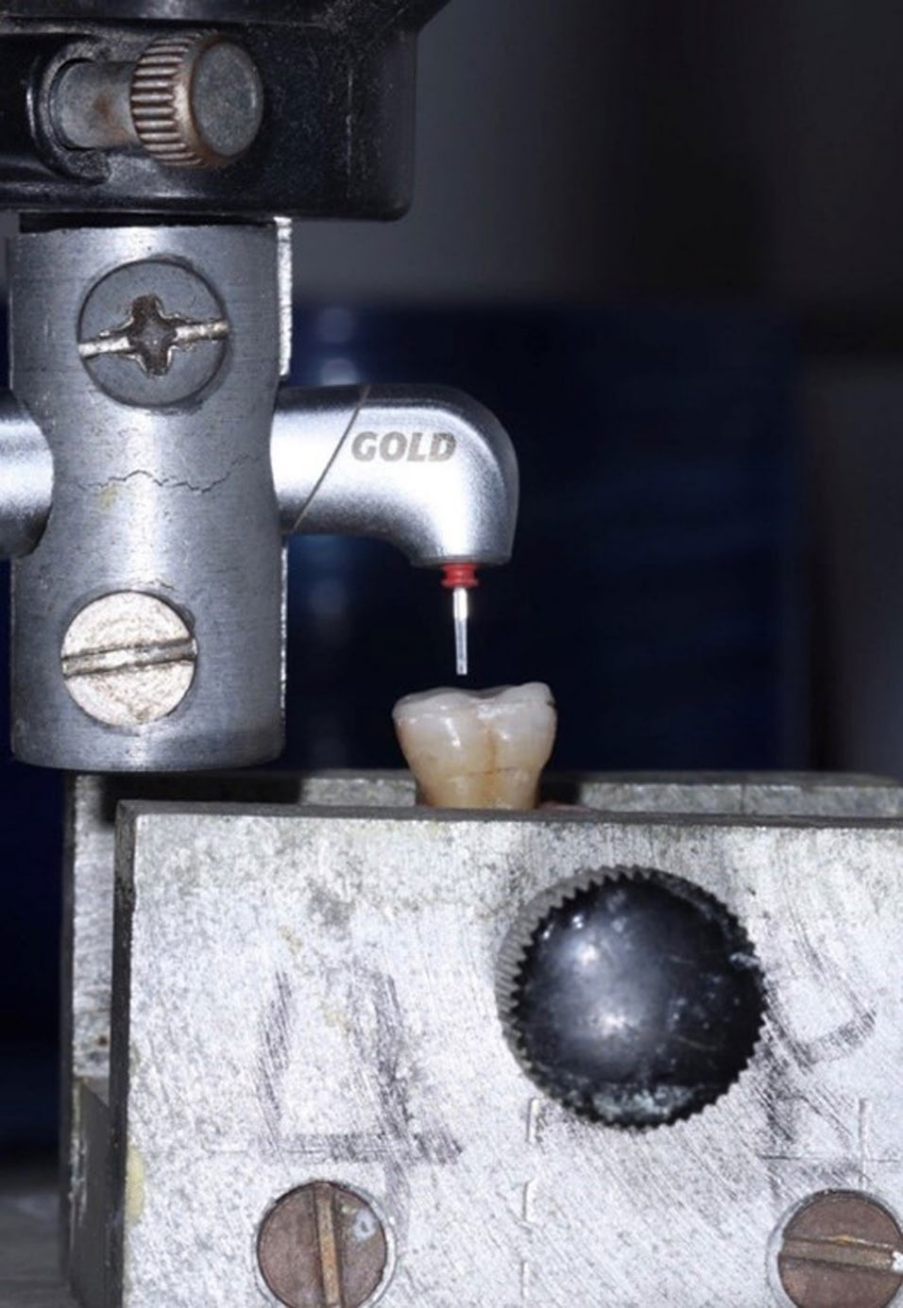
Laser handpiece fixed at a 2 mm distance from the fiber optic tip to the surface of the occlusal veneer

The laser used was Er; Cr: YSGG (Biolase Waterlase iPlus 2.0, USA) with a wavelength of 2780 nm, a power of 6 W, a Frequency 20 Hz, 80% Water, and 60% Air. Gold Handpiece was selected for the study using an MGG6 Saffire tip. An application tip with a diameter of 600 μm was positioned perpendicular to the veneer surface at a 2 mm distance. The distance between the tip and the tooth was standardized using a customized surveyor (Fig. 2). The energy was applied by the scanning method in a noncontact mode through the surface for 15 s with horizontal movements perpendicular to the surface.

The time taken by the veneer to debond after the LASER application was calculated in seconds. The debonded veneers were inspected for possible cracks under a stereomicroscope (Olympus SZ 40, Japan). The debonded veneers were categorized according to their state after debonding into (intact / cracked / catastrophic failure). The mode of bonding failure was also inspected under the stereomicroscope.

Numerical data are presented as the mean with 95% confidence intervals (CI), standard deviation (SD), minimum (min.) and maximum (max.) values. Normality and variance homogeneity assumptions were confirmed using Shapiro-Wilk’s and Levene’s tests, respectively. Data were normally distributed and were analyzed using one-way ANOVA followed by Tukey’s post hoc test. The significance level was set at *p* < 0.05 within all tests. Statistical analysis was performed with R statistical analysis software version 4.3.2 for Windows.

## Results

### Debonding time

**Fig. 3:**
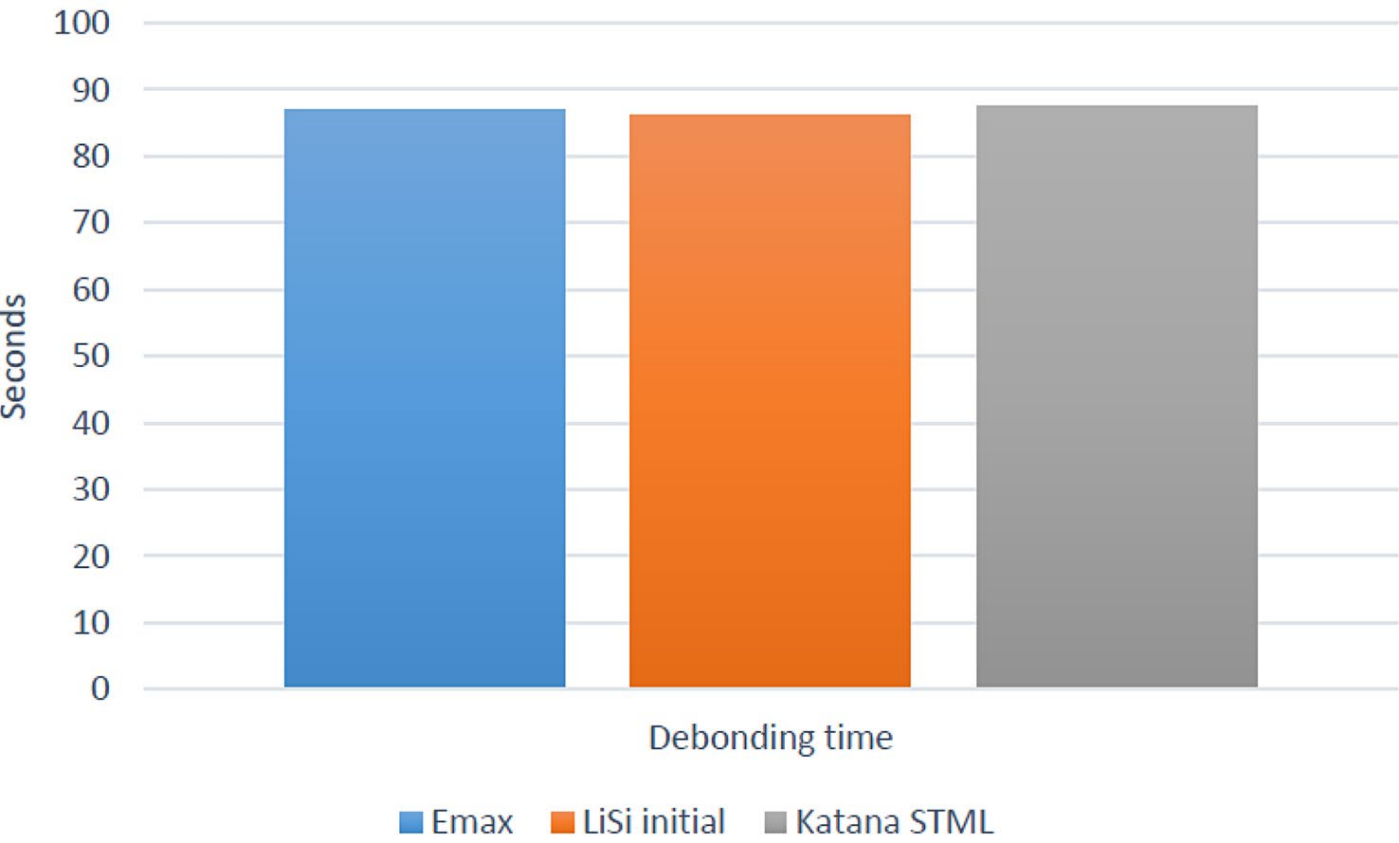
Bar chart showing the average debonding time (seconds)

Descriptive statistics for debonding time (seconds) are presented in Table (1) and Fig. (3). There was no significant difference between the different materials (*p* = 0.995). The longest debonding time was found for Katana STML (87.52 ± 20.45) (seconds), followed by Emax (86.94 ± 20.63) (seconds), while the lowest value was found for LiSi initial (86.14 ± 25.16) (seconds).

**Table 1:** Descriptive statistics for debonding time (seconds)

Group	Mean	95% CI		SD	Min.	Max.
		Lower	Upper			
Emax	86.94	68.86	105.02	20.63	58.80	109.30
LiSi initial	86.14	64.09	108.19	25.16	55.00	123.70
Katana STML	87.52	69.60	105.44	20.45	60.40	108.60

### Debonding status

**Fig. 4:**
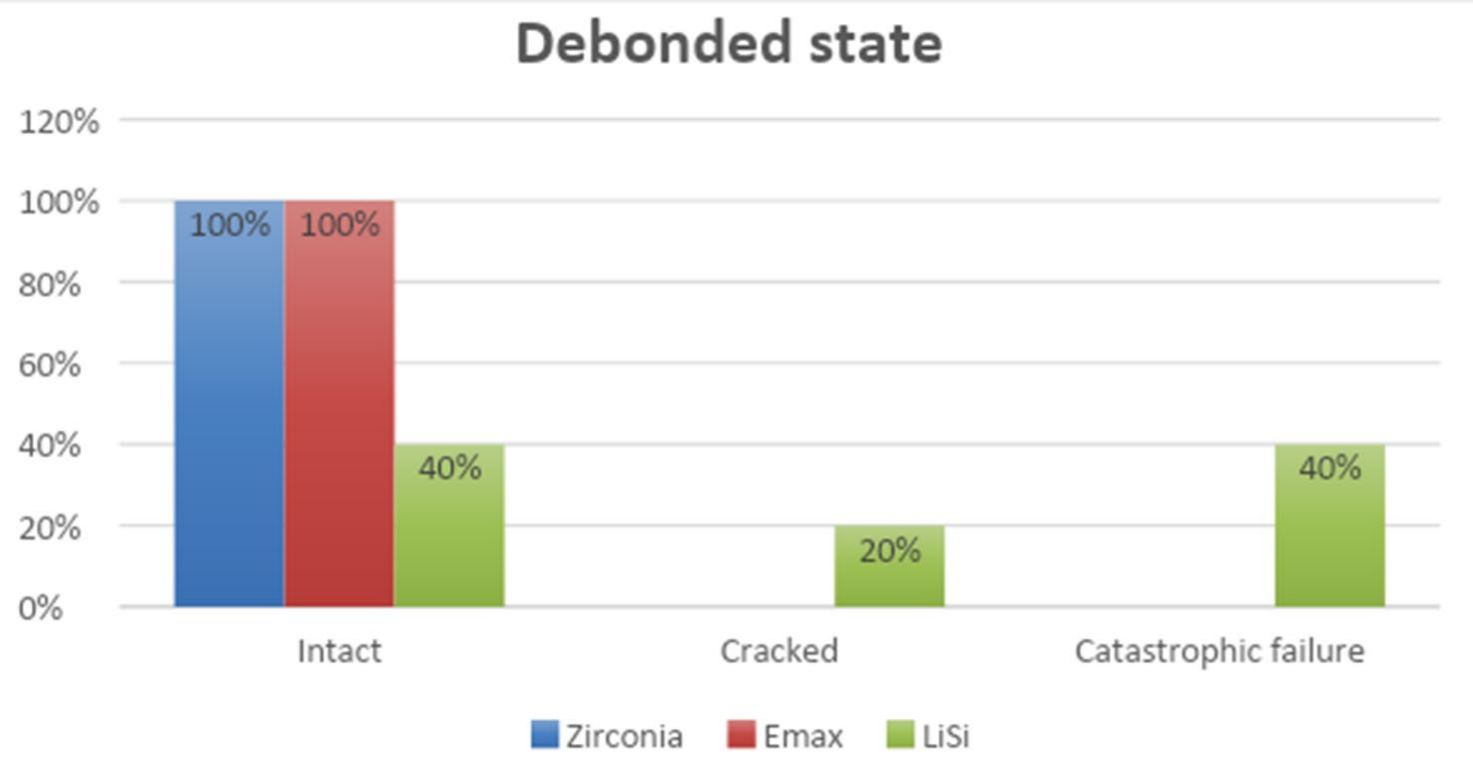
Graph representation of the modes of failure of the 3 materials

**Fig. 5:**
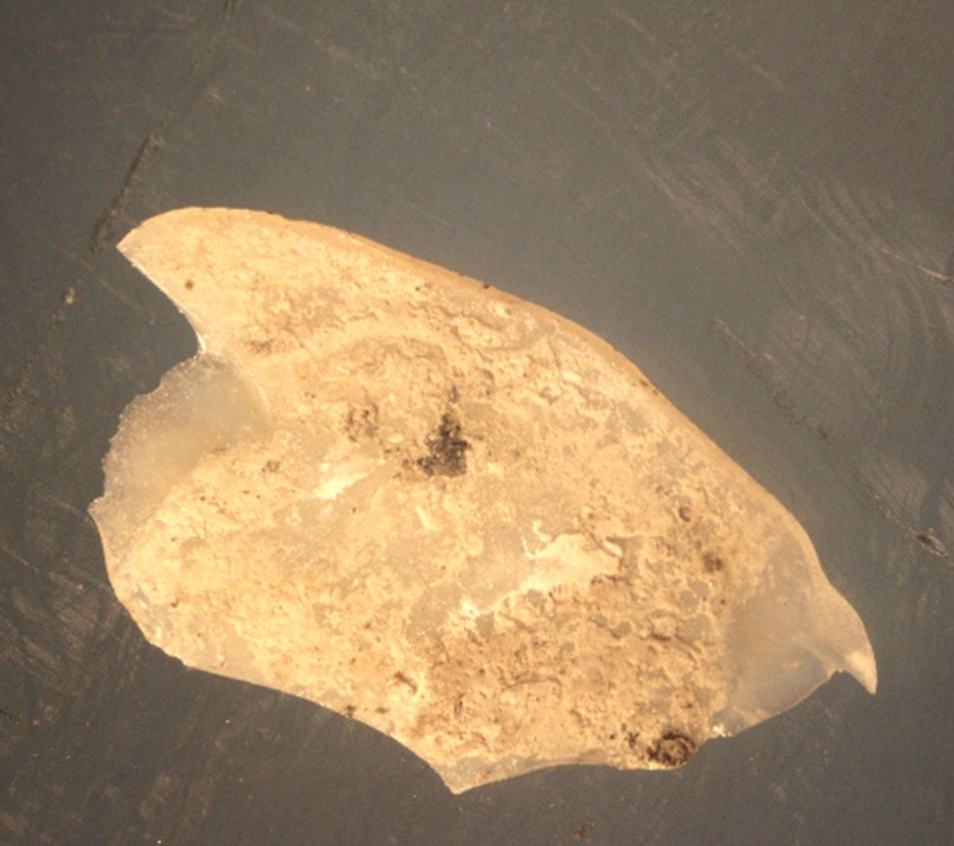
A fragment of a broken debonded LiSi occlusal veneer

**Fig. 6:**
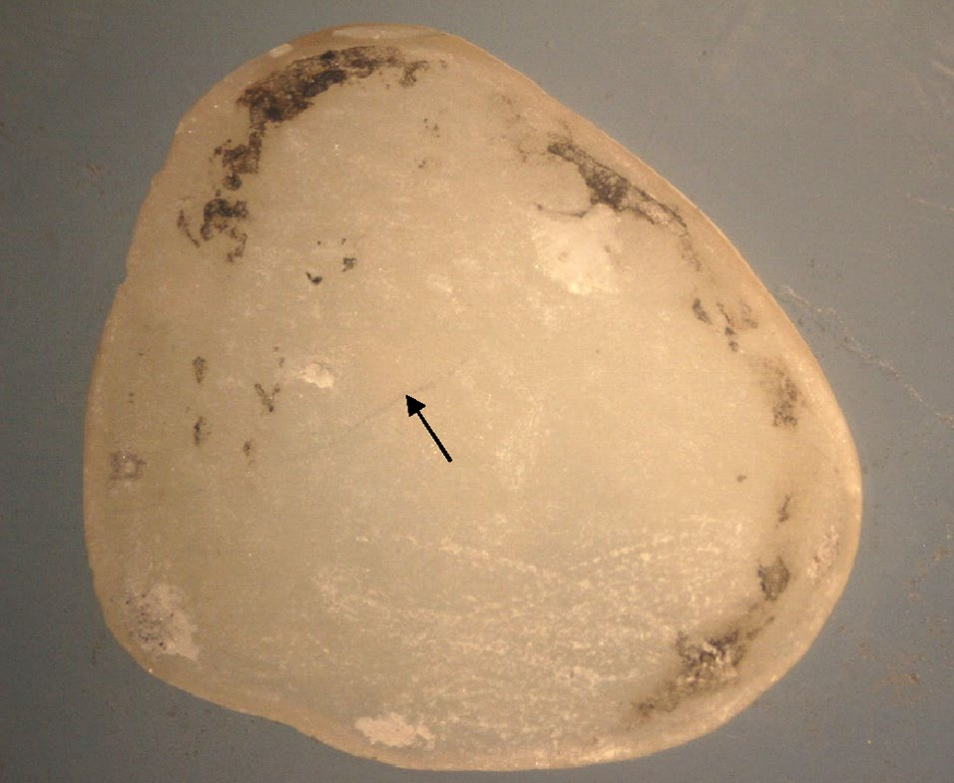
Debonded LiSi occlusal veneer showing a crack

All the Emax and Zirconia samples debonded without damage (Fig. 4). However, only 40% of the LiSi samples were intact after debonding, 40% were fractured (Fig. 5), and 20% exhibited cracks under the stereomicroscope (Fig. 6).

### Mode of failure

None of the samples in any of the three groups exhibited cohesive failure. Two of the Emax samples, 5 zirconia samples, and 1 LiSi sample showed adhesive failure. Six of the Emax samples, 3 of the zirconia samples, and 7 of the LiSi samples showed signs of mixed failure.

## Discussion

Compared to the traditional grinding method, which was used before the introduction of lasers in dentistry, lasers are conservative and comfortable techniques for restoration removal [18]. They also permit the safe retrieval of the restoration for reuse.

Er; Cr: YSGG laser can be efficiently used to remove ceramic restorations. Its wavelength (2940 nm) has a high affinity for water; thus, it easily interacts with resins owing to their water content [23]. Therefore, this type of laser was used in our study.

Clinical reports recommended power settings between 2 and 6 W to remove composite restorations [30]. In this study, the power settings chosen were 6 watts and a frequency of 20 Hz to increase the energy per pulse and decrease the pulse duration so that the cement is rapidly ablated and to avoid thermal softening.

The scanning laser method was used in this study because it produces less heat conduction than does directing the laser to one point [31]. Additionally, air-water spray was used in this study to avoid an increase in the pulp temperature [16, 32]. The materials used in this study are indicated for occlusal veneers because they have mechanical properties suitable for load-bearing areas [4, 33, 34]. 

The bonding of ceramics has been shown to increase their strength, especially in cases where preparation designs provide minimal retention [35, 36]. 

Selective etching was used in this study based on the findings of Krummel et al. [37] who reported that higher fracture resistance was found in the group of occlusal veneers (cusp-fissure thickness of 0.3–0.6 mm) where selective etching was used. Therefore, in a clinical situation where an ultrathin veneer is used selective etching is the technique of choice.

Since optimum bonding protocols of restorations are needed to mimic clinical situations, the surface treatment of silica-based ceramics was done using hydrofluoric acid and a silane coupling agent. For the zirconia samples, air-particle abrasion and MDP primer were used before the resin cement (“APC concept”) [38]. 

Different types of ceramics affect the debonding procedure. Additionally, different shades and translucencies affect laser transmission and, accordingly, the debonding process [20, 24, 25]. In our study, the same shade was used for all the materials. Low translucency blocks were used for the Lithium disilicate and the LiSi initial samples. The choice of STML Katana zirconia was based on its translucency parameter being the closest to Emax while having mechanical properties indicated for posterior teeth. UTML Katana had closer translucency parameters, but since it was the weakest among zirconia materials, it was not used in this study [39, 40]. 

The veneer thickness was standardized for all the materials used. It was reported in the literature that the highest transmission ratio was determined for ceramic veneers 0.5 mm thick (88%). Therefore, it was the thickness of choice.

Johnson et al. [41] replicated the worn occlusal table of teeth with a preparation that left exposed dentin centrally and peripheral enamel. The same design was used in this study.

Freshly extracted teeth are the most suitable substrates for in vitro studies. Since time was taken to collect enough teeth for this research, the extracted teeth needed to be placed in a storage solution to prevent dehydration.

Specimens stored in saline solution had the highest Ca levels [42]. Therefore, the extracted teeth used in this study were stored in a saline solution until preparation.

The laser parameters used were in accordance with those used by Eid et al. [43] in 2021, who used an Er; Cr: YSGG laser using an MGG6 Saffire tip 600 μm in diameter positioned perpendicular to the veneer surface at a distance of 2 mm. Energy is applied by the scanning method through the surface for 15 s with horizontal movements perpendicular to the surface. The laser was applied at a power of 6 W.

In 2004, Van As et al. [42] recommended a power setting for the Er; Cr: YSGG laser of 1 to 3 W for caries, bone, and soft tissue, 2 to 5 W for dentin, and 4 to 8 W for enamel. This seems to support the power setting for removing cement on dentinal surfaces.

Clinical reports recommended medium power settings between 2 and 6 W to remove composite restorations [30]. 

Regarding **debonding time**, our study revealed no significant difference between the types of ceramic materials used in this study. This may be attributed to the similar translucency parameters and the low thickness used in the three materials, which results in the highest transmittance [26]. The similar translucency of the ceramics used allowed for similar transmittance which would result in similar degree of conversion of the resin cement [44]. 

The results of our study are in accordance with those of Alikhasi et al. [27] whose results showed no significant difference in debonding time between feldspathic ceramic and lithium disilicate.

One of the factors influencing debonding time is the volume of cement used. The more cement used, the longer it takes for laser deboning to occur [19]. The marginal accuracy of the restoration can affect the volume of cement used consequently affecting the debonding time. Marginal accuracy is affected by many factors including the milling process [45]. Significant differences in marginal fit were observed between three materials milled using a 3-axis milling machine [45]. Another study compared the marginal fit of lithium disilicate and zirconia using a 3-axis milling machine and a 5-axis milling machine. The results showed a better marginal fit when a 5-axis milling machine was used [46]. 

In our study, the samples were milled using a 5-axis milling machine. This promotes a more accurate marginal fit that results in less cement volume. Other factors that may affect the marginal accuracy include the preparation design and the scanning method used [45]. In our study, the design was standardized, and the same scanner was used in all groups. The previously mentioned factors can help explain the debonding time obtained in our study.

The chemical compositions of the lithium disilicate and LiSi blocks are the same except for LiSi having evenly dispersed, microsized lithium disilicate crystals in the glassy matrix instead of the random dispersion of traditional large-sized crystals in the glassy matrix of the lithium disilicate blocks. Therefore, the current study showed that regarding laser transmission both materials behaved the same and the HDM technology used in the LiSi blocks had no effect on this aspect.

Very few studies have investigated debonding zirconia restorations using laser irradiation, especially studies designed to compare the debonding time between zirconia and other ceramic materials. Rechmann et al. [20] showed that there were differences in the laser transmission between the materials used (lithium disilicate, and zirconia) with the zirconia samples showing the least transmission. However, the zirconia material used was of medium opacity while that of lithium disilicate was of low translucency. Therefore, the differences in the translucency parameters of the materials may have influenced the results.

Our study results contradicted the conclusion of Sari et al. [30] and Eid et al. [43], who reported differences in debonding times between the materials they used. However, the contradiction between these two studies and our study can be attributed to the use of materials with similar transmission values. Additionally, the thickness of the veneers used was 0.5 mm which is reported in the literature to have the highest transmission values. Additionally, the different laser types used in these studies may have contributed to the different results.

Regarding the **state of the debonded samples**, all the lithium disilicate and zirconia samples showed no clinical or microscopic damage. However, most of the LiSi samples exhibited some damage (cracks or fractures). These findings can be explained in light of the differences in the crystal arrangements of the LiSi material.

Kursoglu et al. [47] reported over destruction and weakening of the surface in scanning electron microscope images of specimens on which the Er; Cr: YSGG laser was applied.

The same pattern of dissociation was reported by Gökçe et al. [48] as a possible explanation for the inverse relationship between the shear bond strength (SBS) of the samples and the laser powers at which they were etched. The SBS of the lithium disilicate samples decreased as the laser power used for etching increased. This finding has 2 possible explanations:

1- The high laser power resulted in a heat-generated layer that was poorly attached to the substrate.

2- High laser powers disintegrated crystals.

The second explanation could explain the results of our study.

Considering the previous explanation, the smaller-sized crystals and their dispersion in the glassy matrix could be the reason for the damage that was observed in most of the LiSi samples in this study (unlike the sound lithium disilicate samples).

Zhang et al. [28] tested the effect of an erbium laser (Er: YAG) during debonding on the mechanical and optical properties of dental ceramics using 3 powers (3 W, 4 W, and 5 W). They found that at 5 W, perceptible color changes occurred due to microcracks. These findings shed light on the possible damaging effects of high-power erbium lasers on lithium disilicate ceramics. The zirconia samples showed no damage, which is consistent with other studies performed to debond zirconia using erbium lasers [19, 49, 50]. 

## Conclusions

1- Er; Cr: YSGG was efficient at debonding occlusal veneers made from three ceramic materials (lithium disilicate, highly condensed lithium disilicate, and translucent zirconia).

2- The debonding time did not drastically differ among the different materials used within the 0.5 mm thickness.

3- The Er; Cr: YSGG laser is safe for debonding lithium disilicate and zirconia restorations. However, its effect on LiSi restorations needs further investigation.

## Data Availability

The datasets used and analysed during the current study are available from the corresponding author upon reasonable request.

## Abbreviations

CAD/CAM: Computeraided design/ Computer-aided manufacturing
Er:YAG laser: Erbiumdoped yttrium aluminum garnet laser
Er;Cr:YSGG laser: Erbium, chromiumdoped yttrium, scandium, gallium garnet laser
STL: Standard Tessellation Language
HF: Acid hydrofluoric acid
MDP: 10-methacryloxydecyl dihydrogen phosphate
APC: Airparticle abrasion, zirconia primer, and adhesive composite resin
STML: Super Translucent Multilayered
UTML: Ultra Translucent Multilayered
HDM: High-density Micronization
SBS: Shear bond strength

## Footnotes

2 Morford, Cynthia K., et al. “Er: YAG laser debonding of porcelain veneers.” Lasers in surgery and medicine 43.10 (2011): 965–974.

3 Faul, Franz, et al. “G* Power 3: A flexible statistical power analysis program for the social, behavioral, and biomedical sciences.” *Behavior research methods* 39.2 (2007): 175–191.
